# Deciphering Competitive Interactions of Natural Organic Matter Components at Metal Oxides: Insights from Experiments and Modeling

**DOI:** 10.1021/acs.est.5c00782

**Published:** 2025-10-30

**Authors:** Yun Xu, Tjisse Hiemstra, Yilina Bai, Wenfeng Tan, Liping Weng

**Affiliations:** † Soil Chemistry and Chemical Soil Quality Group, 4508 Wageningen University & Research, Wageningen 6708 PB, The Netherlands; ‡ State Environmental Protection Key Laboratory of Soil Health and Green Remediation, College of Resources and Environment, 534803 Huazhong Agricultural University, Wuhan 430070, China; § Chongqing Academy of Agriculture Sciences, Chongqing 40000, China; ∥ Agro-Environmental Protection Institute, Ministry of Agriculture, Tianjin 300191, P. R. China

**Keywords:** competitive adsorption, molecular fractionation, LCD_cc_ model, heterogeneity, adsorption volume, iron oxide

## Abstract

Natural organic matter (NOM) is a heterogeneous mixture, including humic acid (HA) and fulvic acid (FA), that competitively interacts with metal (hydr)­oxides. Despite its environmental importance, this competition has not yet been measured extensively, and mechanistic modeling is lacking. The present work examined the competitive adsorption to goethite and the corresponding molecular fractionation of HA and FA using UV–vis spectroscopy, acid precipitation, and size exclusion chromatography (SEC). Our findings reveal that on a mass basis, FA particles effectively remove HA particles from the surface. This efficiency can be mainly attributed to an interfacial space limitation in which FA restricts HA adsorption, as evidenced by mechanistic modeling with the Consistent Competitive Ligand and Charge Distribution (LCD_cc_) approach for the heterogeneous adsorption of NOM. The adsorbed FA particles occupying part of the surface prevent HA from accessing the corresponding double-layer space, disproportionally reducing HA adsorption. This restriction leads to a high HA/FA mass exchange ratio (∼2.4 ± 0.6), consequently affecting the mobility and transport of oxyanions (arsenate and phosphate) in the environment. The difference in the partitioning of NOM is also relevant for soil carbon sequestration via the selective preservation of NOM by association with oxide minerals.

## Graphical abstract

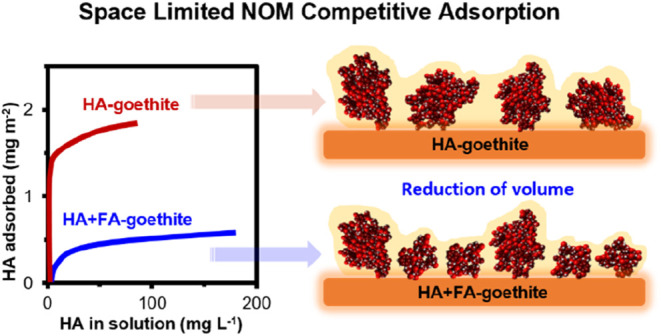

## 1 Introduction

Natural organic matter (NOM) is ubiquitous in various soil and water environments. Its interaction with soil minerals facilitates the formation of mineral-associated organic matter (MAOM) that may shield NOM from decomposition resulting from adsorption, occlusion, aggregation, redox reactions, and polymerization.^1–4^ Among soil minerals, metal (hydr)­oxides, especially iron oxides, are proposed as important soil minerals adsorbing NOM, which plays a significant role in soil organic carbon (SOC) storage and global C-cycling.^5–7^ However, not all components in NOM have equal capabilities to be stabilized by oxide minerals, leading to variation in the MAOM composition. Although the relationships between specific organic compounds and iron oxides have been explored, understanding the competitive interactions of diverse classes of NOM with iron oxides remains a challenge.^8^ Addressing this knowledge gap is critical for uncovering the mechanisms driving MAOM formation and also has important implications for the fate and bioavailability of a series of environment-relevant compounds such as arsenate and phosphate.^9,10^

NOM consists of organic particles that differ in physicochemical properties (e.g., functional group density, molar mass, and size).^11,12^ In attempts to develop a better understanding of the properties of this heterogeneous and complex material, NOM has been separated into various fractions, such as hydrophobic neutrals, hydrophilic acids, fulvic acid (FA), humic acid (HA), and humin.^13,14^ Key differences among these fractions include solubility, molecular size, charge density, and specific ion adsorption.^15–17^ FA and HA are important functional entities of NOM in soil and are known for their high reactivity, particularly concerning the binding of metal ions and association with mineral surfaces; therefore, they have received great attention from researchers.^14,18–20^

A major difference between HA and FA is their molar mass and particle size.^21^ These properties influence the layer thickness of adsorbed NOM and thus the maximum adsorption at the surface of minerals such as metal (hydr)­oxides. For instance, relatively large HA particles (e.g., ∼3 nm) have a maximum adsorption of ∼3 mg m^–2^ on goethite, while relatively small FA particles (∼1 nm) have a maximum adsorption of just 1 mg m^–2^ or less.^22^ Due to the smaller size, FA particles, on average, are located closer to the surface than the larger HA particles. Another difference between HA and FA is the charge density. FA particles usually have a higher density of functional groups and charge than HA particles.^16,23^ The combination of both properties (i.e., molar mass and charge density) affects the interaction of both classes of NOM with the oxide minerals. In general, both are simultaneously present in nature, where they may bind competitively to metal (hydr)­oxide surfaces. Mutual interaction of these contrasting NOM fractions will affect the capacity of oxide minerals to store organic carbon as well as the composition of NOM associated with minerals,^24,25^ thereby impacting the partitioning and transport of a wide range of pollutants and nutrients in the environment.^9,26–28^

Despite their coexistence in soil and water, studies of the interaction of NOM with metal (hydr)­oxides often use either HA or FA only,^10,14,18^ while hardly any research is available regarding the competition between both classes of NOM, nor the ruling factors. One of the main reasons for this knowledge gap is the difficulty in distinguishing these two fractions after they interact with minerals due to the molecular fractionation of HA and FA upon adsorption. This complicates data collection and interpretation. Recently, Xu et al.^15^ have developed a novel methodology for quantifying the concentration of HA and FA in a mixture, which is also applicable to samples after interacting with minerals. This method will now be applied in the current study for the first time to elucidate the competitive interaction of HA and FA in their binding to a metal (hydr)­oxide (goethite), focusing on the factors that determine the overall carbon surface loading.

Surface complexation modeling (SCM) is a useful tool for understanding the (competitive) interactions between adsorbates (e.g., organic acids, oxyanions) and reactive surfaces (e.g., minerals).^22,29,30^ However, to the best of our knowledge, no competitive mechanistic model exists to simulate the simultaneous binding of different NOM components, such as HA and FA, to metal (hydr)­oxides. In this study, we extend the Ligand and Charge Distribution (LCD) model,^31^ which combines advanced surface complexation models such as Non-Ideal Competition Adsorption (NICA) model,^32^ the Charge Distribution MUlti-SIte Complexation (CD-MUSIC) model,^33^ and the ADsorption and AdaPTation (ADAPT) model,^31^ to interpret the competitive adsorption of HA and FA on goethite. This extension incorporates key factors such as multiple modes of heterogeneity, fractionation, and conformational change.^34^ This approach will allow us to account for the contrasting properties of HA and FA and to better understand the factors influencing their competition for binding sites on metal (hydr)­oxides.

The objective of the present work is to investigate the competitive adsorption of two classes of NOM (i.e., HA and FA) differing in size, sites, and charge to metal (hydr)­oxides experimentally and to understand competitive adsorption mechanisms using modeling. Well-defined goethite will be used as a proxy for the metal (hydr)­oxides. The recently established methodology for quantitative analysis of the concentration of both NOM classes in a mixture will be applied to measure their competitive adsorption.^15^ The collected data will be interpreted with the LCD framework for consistent competitive adsorption (LCD_cc_ model).^34^ The insights gained in the present study are helpful to further understand the stabilization mechanisms of different fractions of NOM via organo-mineral associations in soils. Moreover, the results are also relevant to better understand and predict the fate of environmentally important ions, particularly oxyanions (e.g., phosphate, arsenate, and chromate), given that both classes of NOM compete differently with these anions.

## 2 Materials and Methods

### 2.1 Materials

For the batch adsorption experiments, fulvic acid (FA), humic acid (HA), and goethite were used. FA and HA were extracted and purified from the B-horizon of podzol forest soils in The Netherlands following the protocols of the International Humic Substance Society (IHSS). The soil samples were collected from the Telefoonweg in Renkum, The Netherlands (N 52.00982°, E 005.76634°), and the Tongbersven Forest near the village of Oisterwijk, Tilburg, The Netherlands (N 51.34500°, E 005.14413°), respectively. Further details on the physicochemical properties of FA and HA can be found in the Supporting Information (Tables S1, S2, and Figure S2). Goethite was prepared following the same procedure described by Filius et al.^35^ The BET-N_2_ surface area of this goethite is 96 m^2^ g^–1^ and the pristine point-of-zero charge (PZC) is 9.3.

### 2.2 Adsorption Experiments

The competitive adsorption of HA and FA to goethite was measured in two series of batch adsorption experiments. In the first series (Exp I), adsorption isotherms of HA were measured at varying HA concentrations and a constant FA concentration, while in the second series (Exp II), FA adsorption isotherms were measured at varying FA concentrations and a constant HA concentration. For both experiments, the adsorption was studied for pH 4.0 and 6.0 with 1.0 g L^–1^ goethite at a background electrolyte of 0.01 M NaCl. These environmentally relevant pH values reflect typical soil and sediment conditions and capture significantly different charge properties of both goethite and NOM, facilitating a comparison of their adsorption behaviors.

HA and FA were added as a mixed solution to a suspension containing 0.03 g of goethite and brought to a total volume of 30 mL by subsequently adding background electrolyte (NaCl), acid or base solutions (0.1 and 0.01 M HCl and NaOH, respectively), and ultrapure water. For the first series (Exp I), the added FA concentration was 0 or 100 mg L^–1^, and HA concentrations varied from 20 to 240 mg L^–1^. For the second series (Exp II), the total HA concentration was 0 or 160 mg L^–1^ and the FA concentrations varied from 25 to 200 mg L^–1^. These concentration ranges were selected based on previous studies^31,36^ to ensure environmental relevance and comparability with earlier adsorption work and to adequately capture the competitive behavior between HA and FA. It is worth noting that the concentrations of HA and FA refer to their total concentrations. After adsorption, the remaining concentrations in solution were mostly <25 mg L^–1^ DOC, a common range for environmental samples. Blank samples without HA or FA and control samples without goethite were prepared, as well. To minimize the influence of CO_2_, the filling procedure was conducted under moist N_2_ gas. The samples were shaken for 7 days in the dark at 22 °C. The pH of the suspension was checked during the first 24 and 48 h and readjusted to the target value again if necessary, and the final pH was measured after 7 days. The suspensions were centrifuged at 18,000*g* for 30 min and the supernatant was filtered through a 0.22 μm membrane filter (filtration recovery data are provided in Figure S3). The collected filtrates were used for HA and FA quantification and molecular size analysis. The adsorption experiments were conducted in duplicates.

### 2.3 HA and FA Quantification in Mixtures

In single-component samples with only HA or FA, the concentration of HA or FA in the solution was determined by measuring the organic carbon content with a TOC analyzer (Sievers M9, GE). For mixtures of HA and FA, the acid precipitation method and the UV–vis spectroscopic method were used to quantify HA and FA,^15^ and the mean values measured with the two methods were taken as the final concentrations of HA and FA in the solution. The details of both methods have been described in our previous work^15^ (see the Supporting Information). For all of the adsorption experiments, the amount of HA and FA adsorbed was calculated as the difference in HA and FA concentration between the initially added amount and the measured concentration in the supernatant at the end of the adsorption experiment.

### 2.4 Size Exclusion Chromatography (SEC) Measurements

The molecular size distribution of HA, FA, and their mixtures before and after adsorption was investigated with the analysis of size exclusion chromatography (SEC) and UV light absorption (SEC-UV), using a Biosep-SEC S 2000 column (pore size 150 Å, Phenomenex). Details are given in the Supporting Information.

## 3 Modeling Theory

In the current work, the recently developed Consistent Competitive Ligand and Charge Distribution (LCD_cc_) model^34^ was used and further developed to describe the competitive adsorption of HA and FA to goethite. The model combines the advanced surface complexation models developed for NOM (NICA) and metal (hydr)­oxides (CD-MUSIC) to describe reactions on oxide surfaces covered with NOM. The latest version of the model (LCD_cc_) also accounts for other factors active in the adsorption process, including multiple modes of heterogeneity, change in molecular conformation of adsorbed particles, and size fractionation. These key features of LCD_cc_ are important for modeling competitive HA and FA adsorption to oxides, which are illustrated below.

### 3.1 Multiple Modes of Heterogeneity and Variation in Affinity

In the LCD_cc_ model, HA and FA are considered heterogeneous particles varying in numbers of functional groups, particle charge, and molar mass and size, resulting in a distribution of affinities for binding to metal (hydr)­oxide surfaces. In the LCD_cc_ model, the role of heterogeneity on their adsorption to oxides is accounted for at both the particle and ligand levels.

At the particle level, particles may vary in terms of molar mass and site density, resulting in variations in adsorption affinity to oxides, even within one NOM fraction (i.e., HA or FA). The influence of particle-level heterogeneity on HA or FA adsorption is implemented in the expression for the adsorption equilibrium between the solution and the adsorbed HA or FA particles at the surface, known as the Langmuir–Freundlich (LF) equation:

1 $${\frac{\phi_{\mathrm{ads}}}{1-\phi_{\mathrm{ads}}}={(\mathrm{K̃}}_{o}\phi_{\mathrm{sol}})}^{q}$$

in which *ϕ*_sol_ and *ϕ*_ads_ represent the concentration of HA or FA particles in the solution and adsorbed phase respectively, both defined as a volume fraction (m^3^ m^–3^). The median value for the affinity is given as *K̃*_o_ and the width of the affinity distribution related to particle-level heterogeneity is expressed in coefficient *q*. A value of *q* = 1 corresponds to a homogeneous particle distribution, whereas 0 < *q* < 1 indicates increasing heterogeneity in particle properties such as particle size or molar mass and surface functional group (e.g., carboxyl group) densities.^34,37^ Details of the sensitivity analysis for the particle-level heterogeneity parameter (*q*) are provided in the Supporting Information.

For HA used in the present study, the particle-level heterogeneity has been probed previously with phosphate, revealing a parameter value of *q* = 0.1.^34^ Following a similar approach, a value of *q* = 0.3 is found for FA^37^ (see the Supporting Information). The higher value of *q* indicates less variation in the affinity among FA particles compared with HA, showing that FA is less heterogeneous at the particle level than HA.

The challenge of applying the seemingly simple adsorption equation (eq 1) is to derive the median affinity *K̃*_o_. This affinity (*K̃*_o_) is not a constant but varies with the solution conditions and the surface loading, expressing the overall change in the energy states of HA or FA in the solution and the adsorbed phase. In the LCD_cc_ model, the value of *K̃*_o_ is calculated with the ADAPT module in combination with the NICA and CD-MUSIC module for HA or FA, respectively, based on an average molar mass (*M*_w_) for each of them. The chemical heterogeneity of the functional groups at the ligand level is dealt with by the NICA equation, which assumes a continuous distribution of binding affinity of these groups toward ions and surface sites of oxides. Detailed information regarding the adsorption energy change and adsorption affinity calculation is provided in the Supporting Information.

### 3.2 Interfacial Change of Molecular Conformation

In the LCD_cc_ model, the spatial distribution of adsorbed NOM particles (*f*_0+1_, *f*_1+2_, *f*_d_) is one of the key factors in calculating the adsorption affinity and the effects of adsorbed NOM on the binding of other adsorbates. This distribution is assumed to be dynamic, reflecting the flexible and soft nature of NOM. Specifically, the arrangement of adsorbed NOM is regulated by the gradients of the electrostatic potentials in the Stern layers. Due to the strong interaction of the functional groups of NOM (HA and/or FA) particles with the surface, NOM particles first occupy the Stern layers up to a certain maximum (*Γ*_MST_) that is generally lower than the physical maximum (*Γ*_MST_^o^), both in mg m^–2^.^38^ Adsorbed particles in excess will be present outside the compact part (Stern layers) of the electrical double layer (EDL). The resulting interfacial distribution is defined with

2a $$f_{0+1}+f_{1+2}+f_{d}=1$$

2b $$f_{d}=\left(\Gamma_{\mathrm{tot}.}-\Gamma_{\mathrm{MST}}\right)/\Gamma_{\mathrm{tot}.}\;\mathrm{for}\;\Gamma_{\mathrm{tot}.}>\Gamma_{\mathrm{MST}};\;f_{d}=0\;\mathrm{for}\;\Gamma_{\mathrm{tot}}<\Gamma_{\mathrm{MST}}$$

2c $$\theta_{s}=\Gamma_{\mathrm{MST}}/\Gamma_{\mathrm{MST}}^{o}$$

2d $$R=f_{0+1}/\left(f_{0+1}+f_{1+2}\right)$$

where *f*_0+1_ and *f*_1+2_ are the fractions of the functional groups on NOM particles that occupy the Stern layers, distributing the corresponding charge over the 0- and 1-plane and the 1- and 2-plane, respectively. Excess adsorbed NOM particles (*f*_d_) are attributed to an additional electrostatic plane for adsorption (d-plane), beyond the Stern layers, to which also the electrolyte ions, present in this part of the EDL, are attributed, as given in the Supporting Information (Figure S6). In the LCD_cc_ model, the maximum Stern layer occupation (*θ*_s_), relative to its physical maximum (eq 4), and the relative fraction (*R*) residing in the first Stern layer (eq 5) are regulated by the gradients of the electrostatic potential within the compact part of the EDL.^34^ These potential gradients are employed in functionals (eq S5 in the Supporting Information) to calculate the iterative spatial distribution of HA and FA during competitive adsorption.

### 3.3 Size Fractionation

To calculate the median affinity *K̃*_o_ in eq 1 using the ADAPT approach, the mean molar mass (*M*_w_) of adsorbed HA and FA is needed. Due to the polydisperse nature of NOM and the variation in the affinity of the particles for adsorption to mineral surfaces, particles with a higher affinity will be preferentially adsorbed, leading to a change in the mean molar mass (*M*_w_) of the adsorbed NOM. Our earlier investigation on HA adsorption has found that the *M*_w_ (kDa) changes as a function of its relative adsorption.^38^

3 $$M_{w}=M_{o}+k\left(1-\frac{\rho_{\mathrm{ssr}}C_{\mathrm{HA}}}{{\mathrm{HA}}_{\mathrm{tot}}}\right)$$

where *ρ*_ssr_ is the solution-to-solid ratio (i.e., 1.0 L g^–1^ goethite), *C*_HA_ (mg L^–1^) is the HA concentration in solution, and HA_tot_ (mg g^–1^) is the total amount of HA added. In eq 6, *M*_o_ is the molar mass of the most preferred HA particles in the adsorption (*M*_o_ = 1.6 kDa for the HA used in this study) and *k* is the fractionation factor.^38^ The value of *M*_o_ (1.6 kDa) reflects the preferential adsorption of lower-molecular-mass HA components, as *M*_o_ is significantly lower than the average molar mass of the original HA (17 kDa). Eq 6 can be used to describe the fractionation as a function of the HA concentration left in the solution at different values of the solution-to-solid ratio (SSR).

In LCD_cc_ modeling, the description of the fraction is incorporated. The SEC measurements for FA show that the mean molar mass of the adsorbed FA does not undergo significant changes upon adsorption (*k* ∼ 0). Therefore, the molar mass of the adsorbed FA is kept constant in the model calculations and set to *M*_w_ of the original FA (1.8 kDa). Details concerning the size fractionation in competitive adsorption systems are discussed in Section 4.

To facilitate the calculations with the software, the amount of HA adsorbed was calculated by setting FA adsorbed to the measured amount and vice versa. In this approach, a consistent calculation of the interfacial distribution can still be achieved for both components via iterations. The model calculations were implemented in the software ORCHESTRA.^39^ The applied model parameters are summarized in the Supporting Information (Tables S2–S4).

## 4 Results and Discussion

### 4.1 Competitive Adsorption Data

The competitive adsorption of HA and FA at pH values of 4 and 6 was studied in two batch experiments. In the first series (Exp I), HA of different concentrations was added to the goethite suspensions in the presence of a fixed value of FA (100 mg L^–1^). For comparison, the HA adsorption in the absence of FA was also measured. As shown in Figure 1a, with the increase of the HA concentration, the amount of HA adsorbed increased (dark blue symbols), whereas the amount of FA adsorbed decreased (dark red symbols). Compared with HA adsorbed in the absence of FA (light blue symbols), FA addition (100 mg L^–1^) drastically decreased HA adsorption. This is indicated by the red arrow in Figure 1a.

**Figure 1:**
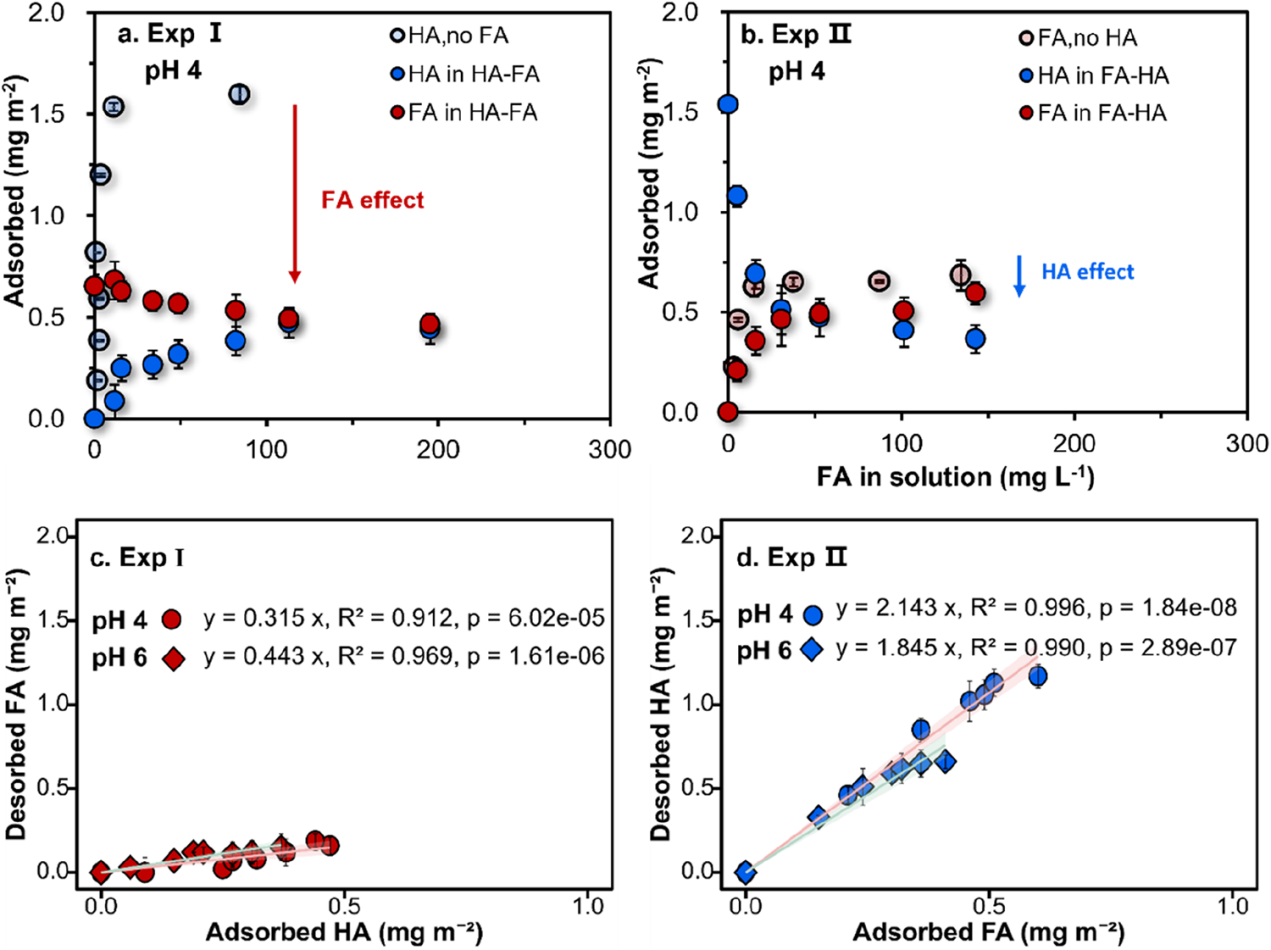
Adsorption isotherms of HA (blue) and FA (red) to goethite (1 g L^–1^) at pH 4, with a background electrolyte of 0.01 M NaCl in Exp I (a) and Exp II (b). Desorbed FA related to adsorbed HA in Exp I (c), and desorbed HA related to the adsorbed FA in Exp II (d) at pH values of 4 (circles) and 6 (diamonds). Exp I has HA additions between 0 and 240 mg L^–1^ at a constant FA addition of 100 mg L^–1^, while Exp II has FA additions between 0 and 200 mg L^–1^ at a constant HA addition of 160 mg L^–1^. The measured adsorption isotherms at pH 6 can be found in Figure 3. Error bars represent the standard deviation of the duplicate measurements. Confidence intervals of the fitted regressions are shaded.

In the second series (Exp II), FA of different concentrations was added to the goethite suspensions in the presence of 160 mg L^–1^ HA (Figure 1b). The increase of the added FA led to a considerable decrease in the HA adsorption (dark blue symbols) at both pH values of 4 and 6. But compared to the single FA adsorption systems (light red symbols), the addition of 160 mg L^–1^ HA decreased the maximum FA adsorbed (dark red symbols) only marginally (<0.1 mg m^–2^) as illustrated with the blue arrow.

The competitive interaction of FA and HA results in a near-linear relation between the adsorption and desorption of both classes of NOM (Figure 1c,d). In Figure 1c,d, the exchange ratio is represented by the slope of the linear regression line, corresponding to the mass of NOM desorbed per unit mass adsorbed. The exchange ratio (mass desorbed/adsorbed) is high (pH 4:2.15 ± 0.05, pH 6:1.83 ± 0.07) when FA is added and HA is desorbed (Exp II), but low (pH 4:0.31 ± 0.03, pH 6:0.44 ± 0.03) when HA is added and FA is desorbed (Exp I). Consequently, the marginal replacement of FA by HA in Exp I leads to almost no increase in the mean molar mass (*M*_w_) of the adsorbed humic particles (Figure S7a), in contrast to the substantial decrease of *M*_w_ in Exp II when FA is added and HA is desorbed (Figure S7b). The exchange ratio on molar mass can be found in the Supporting Information (Figure S8). Additionally, there is no significant difference in the competition or exchange ratio among the studied pH levels (circles and diamonds in Figure 1c,d, respectively). The minor variations between the two pH treatments observed in the mean molar mass (*M*_w_) changes in Figure S7 further support the finding that the competition between HA and FA remained largely unaffected within the pH range of 4 to 6.

Although the measured responses look very different, the results of both competition experiments (Exp I and Exp II) are consistent. Both experiments indicate that, on a mass basis, HA is less competitive than FA in its adsorption to goethite when both are present together. This behavior suggests that FA exhibits a higher surface affinity, likely due to its greater density of carboxylic groups and smaller molecular size, which enable FA particles to occupy surface sites more effectively, thereby limiting HA adsorption (see Section 4.3.2 for discussion). When HA is added, the effect of HA on the adsorbed FA is weak, while on the contrary, when FA is added, about twice as much adsorbed HA can be removed.

### 4.2 Size Fractionation in Binary FA–HA Systems

The mean molar mass (*M*_W_) of adsorbed NOM is a crucial factor in modeling the NOM adsorption, as the molar mass substantially affects the free energy of NOM adsorption,^36,38^ which is used in the ADAPT module of the LCD model to calculate the median adsorption affinity constant (*K̃*_o_) of HA or FA adsorption in eq 1. Due to adsorptive fractionation, the mean molar mass of adsorbed NOM may differ from the value of the added NOM. For single-component HA systems (without FA), the change in molar mass has been studied with SEC, resulting in the mathematical relationship of eq 6.^38^ However, they have not yet been evaluated for competitive HA/FA systems.

In our competitive systems, the mean molar mass of mixed HA and FA (*M*_W_mix_) in the adsorbed phase has been determined experimentally with SEC measurements (Figure 2). This quantity can be used to assess the mean molar mass of HA (*M*_W_HA_) in the adsorbed phase if the mean molar mass of the mixture (*M*_W_mix_) is the weighted sum of the mean molar mass of the adsorbed HA (*M*_W_HA_) and the adsorbed FA (*M*_W_FA_) according to

4 $$M_{W\_\mathrm{mix}}=M_{W\_\mathrm{HA}}\times \varphi_{\mathrm{HA}}+M_{W\_\mathrm{FA}}\times \varphi_{\mathrm{FA}}$$

in which *φ*_HA_ and *φ*_FA_ are the experimental mass fractions of HA and FA adsorbed, respectively, over the total amount of HA and FA adsorbed, and *φ*_FA_ = 1–*φ*_HA_. For deriving with eq 7 the mean molar mass of adsorbed HA (*M*_W_HA_), the mean molar mass of the adsorbed FA is set equal to the value of the original FA (*M*_W_FA_ = 1.8 kDa) because little fractionation is seen in single FA systems (data not shown).

**Figure 2:**
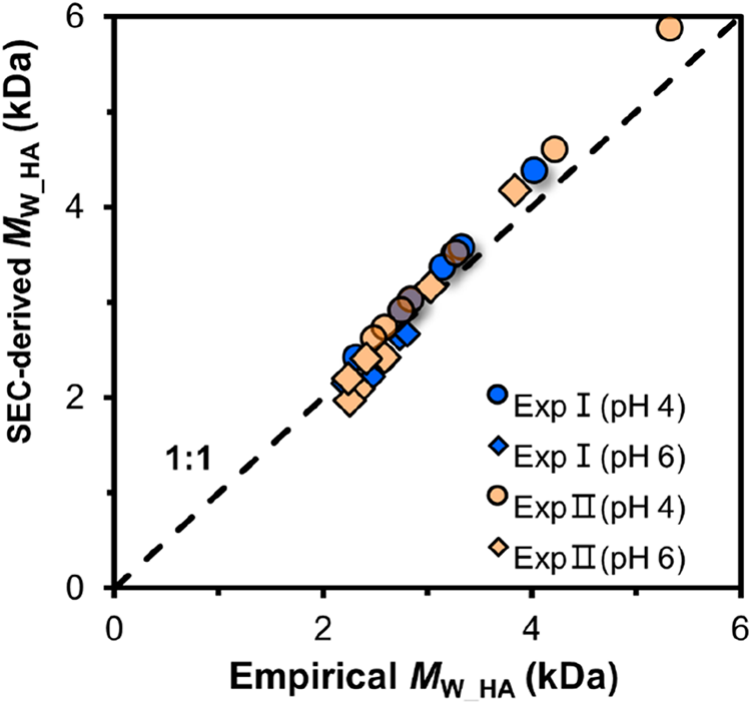
SEC-derived mean molar mass of adsorbed HA (SEC-derived *M*_W_HA_) in the competitive adsorption experiments in comparison to the values derived with an empirical fractionation relation (eq 6) (empirical *M*_W_HA_). Eq 6 has been derived from single HA adsorption results in a previous study.^38^ Data are for pH values of 4 and 6 in the 0.01 M NaCl, 1.0 g L^–1^ goethite systems of Figure 3 (Exp I and Exp II). The dashed line is the 1:1 line.

Figure 2 compares the *M*_W___HA_ values derived from SEC measurement using eq 7 with the values calculated with the empirical fractionation relation (eq 6) for Exp I and Exp II. The mean molar masses of both approaches are in good agreement, suggesting that the empirical relation (eq 6) found in single HA adsorption systems can still be applied to predict the *M*_w_ of adsorbed HA in the presence of FA at the same background electrolyte condition (0.01 M NaCl). In our earlier work,^34^ we have found that this empirical relation is also applicable in the competitive systems of HA with phosphate, indicating that the size selectivity in HA adsorption depends mainly on the fractions of HA adsorbed, irrespective of a large variation in other factors such as the HA loading, pH, and the presence of FA or phosphate, which all lead to the variations in the fraction of HA adsorbed. Given the substantial variability in the molecular composition, size distribution, and functional group content of NOM, the parameters in eq 6 are expected to differ among different NOM materials.

This finding suggests that the affinity of HA particles for binding to oxides varies, and this variation is correlated with the molar mass and particle size. The difference in adsorption affinity among HA particles is the driving force behind the selective adsorption of HA, which behaves consistently under the influence of factors such as pH, HA loading, FA, and phosphate, if present.

The good agreement given in Figure 2 indicates that the adsorption of HA in the presence of FA behaves similarly to that without FA, suggesting that the presence of FA does not influence the molar mass of HA, meaning that HA and FA react as separate entities, without merging. It suggests that organo-organic interactions, as postulated in the multilayer model,^4,40^ do not play a significant role in this adsorption process.

### 4.3 Modeling the Simultaneous Adsorption of HA and FA Particles

The LCD_cc_ model can successfully describe the adsorption of HA in single-component systems in the absence and presence of phosphate.^34^ The model can also successfully describe FA adsorption, as illustrated in the Supporting Information.

Modeling of NOM adsorption in HA and FA competitive systems requires the adaptation of the Langmuir–Freundlich (LF) formulation in eq 1. For HA and FA in a binary system, the distribution of HA or FA particles over the solution (ϕ_sol_) and adsorption (ϕ_ads_) phase results from a simultaneous establishment of two adsorption equilibria, defined as

5a $$\frac{\phi_{\mathrm{HA},\mathrm{ads}}}{1-\sum \phi_{p,\mathrm{ads}}}={\left({{\mathrm{K̃}}_{\mathrm{HA}}\phi }_{\mathrm{HA},\mathrm{sol}}\right)}^{q_{\mathrm{HA}}}$$

5b $$\frac{\phi_{\mathrm{FA},\mathrm{ads}}}{1-\sum \phi_{p,\mathrm{ads}}}={\left({{\mathrm{K̃}}_{\mathrm{FA}}\phi }_{\mathrm{FA},\mathrm{sol}}\right)}^{q_{\mathrm{FA}}}$$

in which the volume fractions of HA and FA adsorbed (*ϕ*_*p*,ads_) are indexed with the letter *p*. In the expressions, the nominator represents the volume fraction of the adsorbed phase occupied by HA or FA, and the denominator represents the unoccupied space of the adsorbed phase expressed as a volume fraction.

In eqs 5a and 9, the volume fractions of the HA or FA particles (*ϕ*_*p*,ads_) can be related to the amounts adsorbed (*Γ*_*p*,ads_ in kg m^–2^) according to

6a $$\phi_{p,\mathrm{ads}}=\frac{\Gamma_{p,\mathrm{ads}}}{V}\frac{1}{\rho_{p}}$$

in which *ρ* *_p_* is the corresponding mass density of HA or FA in kg m^–3^ and *V* is the total volume of the adsorbed phase in m^3^ m^–2^.

In the solution phase, the relation between the volume fraction (*ϕ*_*p*,sol_ in m^3^ m^–3^) and equilibrium mass concentration (*C*_*p*_, kg L^–1^) can be defined with

6b $$\phi_{p,\mathrm{sol}}=C_{p}\frac{1}{\rho_{p}}$$

The mass density of HA and FA particles (*ρ*_*p*_) can vary due to molecular condensation and ranges from 700 kg m^–3^ for well-hydrated HA and FA particles^41^ to 1700 kg m^–3^ for particles in the dry state.^42^ To simplify the model calculation, an in-between value of 1250 kg m^–3^ was applied for HA and FA in both the solution and adsorbed phases. The sensitivity analysis for the mass density can be found in Figure S9.

Physically, the adsorption of NOM will reach a maximum value if the entire surface is occupied. At favorable adsorption conditions, this may occur at the plateau value of the adsorption isotherm. This maximum is very different for both end-members (HA and FA) of our binary systems. This is due to the difference in the molecular size and the resulting adsorption layer thickness and volume. A Stern layer with a thickness of *d*_ST_ ≈ 0.8 nm^43^ can accommodate all functional groups of adsorbed FA in single systems because our FA particles are relatively small (∼1 nm). Correspondingly, the maximum possible adsorption volume for FA can be set to *V* = 0.8 × 10^–9^ m^–3^ m^–2^, which corresponds to *Γ*_MST_^o^ in eq 2. The physical maximum of the adsorption volume of HA has been set to *V* = 3 × 10^–9^ m^–3^ m^–2^; as our HA particles are relatively large (∼3 nm), they can therefore protrude out of the Stern layer, entering the diffuse layer.^36^ Thus, the limiting volumes are different for HA and FA.

The question arises regarding which space volume constraint is the most realistic when HA and FA particles are simultaneously adsorbed in binary systems, i.e., which total volume (*V*) should be used to calculate the volume fraction of adsorbed HA and FA. To address this question, we have evaluated the competitive adsorption of HA and FA, using the same sets of adsorption parameters derived previously for HA and FA, respectively,^34^ but different assumptions regarding the adsorption volume *V* (in m^3^ m^–2^). Two scenarios have been considered, as discussed below.

#### 4.3.1 Scenario 1: Constant Adsorption Volume

In the first set of model calculations, we assumed that the space constraints for HA and FA adsorption in mixed systems are relatively small. The adsorption volume has been fixed to a constant value, equal to the value found for the HA adsorption in single-component systems (*V* = 3 × 10^–9^ m^–3^ m^–2^), equivalent to a layer thickness of 3 nm. This allows a sufficient adsorption space for FA as well. The HA and FA adsorption predicted by the LCD_cc_ model for pH values 4 and 6 using this constant value of *V* are given with dotted lines in Figure 3.

**Figure 3:**
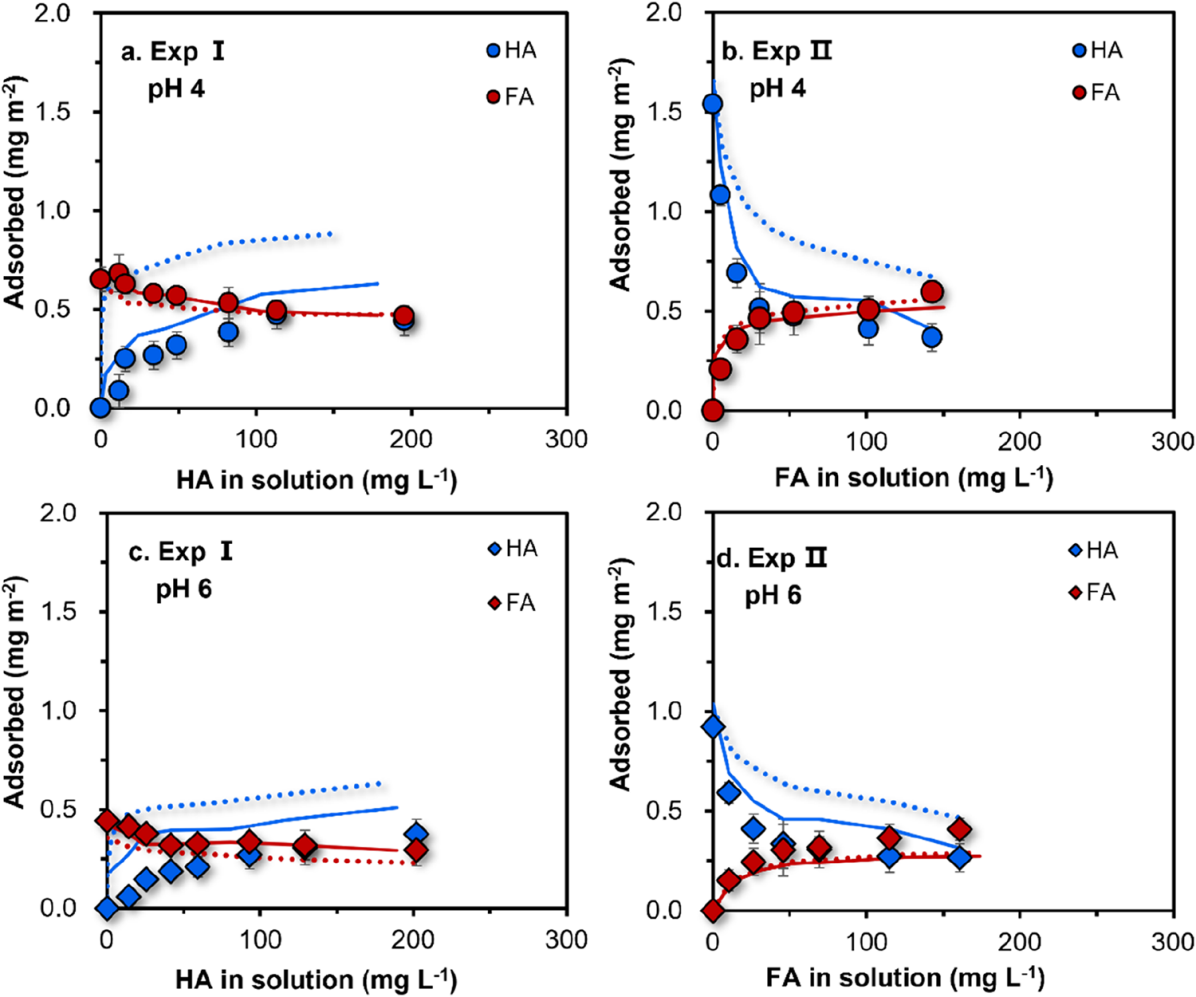
Comparison of the modeled HA and FA adsorption at values of pH 4 (a&b) and 6 (c&d), assuming (1) a constant adsorption volume (Scenario 1, dotted lines) or (2) an adsorption volume proportional to the relative presence of FA and HA (Scenario 2, solid lines). In Exp I, the FA concentration is constant, while HA addition varies, and in Exp II, the HA concentration is constant, while FA addition varies, as detailed in Figure 1. Model performance is evaluated using root mean square error (RMSE): for HA, RMSE decreases from 0.22–0.32 in Scenario 1 to 0.10–0.14 in Scenario 2; for FA, RMSE values remain relatively consistent across both scenarios (∼0.05), indicating a stronger impact of spatial constraints on HA adsorption.

For FA in competition with HA, the model predictions (dotted lines) are in reasonable agreement with the experimental data for the two series of batch experiments (Exp I and Exp II). However, for HA in competition with FA, the predicted adsorption is much higher than that measured, particularly at high loading. This suggests that the adsorption of HA is more limited by space than that assumed in this scenario.

#### 4.3.2 Scenario 2: Variable Adsorption Volume

In the second set of model predictions, by applying the same adsorption parameters, the adsorption volume (*V*) is variable, assuming that the volume is proportional to the fractions of HA and FA in the adsorbed phase.

7 $$V=\sum \omega_{i}V_{i}$$

in which *ω*_*i*_ is the relative volume fraction of the adsorbed HA (*i* = 1) and FA (*i* = 2) in the competitive adsorption. These volume fractions are obtained iteratively during the modeling because they are related to the amounts adsorbed (*Γ*_*p*,ads_ in kg m^–2^) (eq 10). In eq 12, *V*_*i*_ is the volume of the adsorbed phase that can be occupied by either HA (∼3 × 10^–9^ m^3^ m^–2^) or FA (∼0.8 × 10^–9^ m^3^ m^–2^) in single HA or FA systems, respectively.

By inclusion of a variable adsorption volume (eq 12), the predicted adsorption of both HA and FA agrees well with the experimental data (solid lines in Figure 3), as indicated by the lower root mean square error (RMSE) values. Our calculation exercise identifies space limitation as a crucial factor in the competitive adsorption of HA and FA particles, especially for the HA particles. Specifically, the application of the variable adsorption volume model (Scenario 2) resulted in significantly lower RMSE values (0.10–0.14) compared to those obtained using the constant volume assumption (Scenario 1; RMSE = 0.22–0.32), underscoring the importance of spatial constraints in accurately capturing adsorption behavior. Earlier research on the competition between phosphate and HA or FA has indicated that electrostatic interactions dominate the nature of the interactions between HA or FA with phosphate on the goethite surface.^44^ However, the electrostatic potentials calculated when modeling HA adsorption with given FA adsorbed at the 0-, 1-, and 2-planes for both model simulations (constant or variable adsorption volume) do not strongly vary in our experiments (Figure S11), supporting the space limitation as a key factor for the adsorption of HA particles in the competitive systems with FA.

The competition between HA and FA particles is pH- and loading-dependent, as evident from Figures 1 and 3. Both HA and FA adsorb more strongly at a low pH than at a high pH. At lower pH (i.e., pH 4), oxide surfaces exhibit a higher amount of positive charge (due to protonation of surface hydroxyls), which leads to enhanced electrostatic attraction of the negatively charged NOM particles. Conversely, at higher pH (i.e., pH 6), the oxide surface becomes less positively charged, which reduces the electrostatic attraction of NOM. In addition, more deprotonation of the functional groups increases the negative charge of NOM particles, enhancing lateral electrostatic repulsion between adsorbed NOM. At high-loading conditions, HA is strongly affected on a mass basis by the adsorption of FA, as shown by the arrows in Figure 1. This decrease is predominantly due to space limitation initiated by the preferential adsorption of FA. The higher density of functional groups in FA and the smaller size of the FA particles facilitate the formation of inner-sphere complexes with surface sites on oxides through ligand-exchange complexation, resulting in a stronger binding affinity of FA in comparison to HA.^44,45^

The essence of the adsorption volume limitation revealed in the current study can be envisioned as a surface area limitation on oxides. The added FA particles efficiently cover part of the surface, making it inaccessible to HA particles, which can then only occupy the remaining surface with the corresponding double-layer space, and vice versa. Consequently, the volume (in m^3^ m^–2^) available for HA adsorption reduces disproportionally with the increase in adsorbed FA.

## 5 Implications and Outlook

Interactions between natural organic matter (NOM) and minerals have been explored extensively in the literature. However, a comprehensive understanding of the interactions, particularly concerning the regulating mechanisms in the competition of different components of NOM, is lacking. The present study is, to our knowledge, the first attempt to quantitatively describe the interaction of two different types of NOM (HA and FA) with a metal (hydr)­oxide (goethite).

Our study reveals that FA adsorption disproportionally suppresses the total surface carbon storage (mg C m^–2^), as these small and highly charged molecules can effectively confiscate surface area and reduce interfacial space for the adsorption of larger humic particles (e.g., HA). Consequently, less carbon in total can be bound and preserved by direct interaction with metal (hydr)­oxide surfaces. This finding may also suggest that the oxide surfaces in soil are probably covered mainly by FA-like NOM. This type of NOM entity (FA-like) is potentially soluble unless associated with soil minerals, forming organo-mineral associations, or forming a supramolecular structure with other NOM entities (e.g., HA).

The interaction of different classes of NOM with a metal (hydr)­oxide is also highly relevant for the environmental fate of oxyanions (e.g., phosphate and arsenate), as various types of NOM particles may compete differently with oxyanions^44^ (as illustrated in the Supporting Information). In the case of HA, particularly at a high surface loading, a substantial proportion of the functional groups reside outside the compact region of the electrical double layer (EDL). Conversely, FA particles bind primarily close to the surface and have a higher charge density. Consequently, FA competes much more strongly than HA for the binding of oxyanions, leading to the mobilization of these ions.

The competitive LCD_cc_ model framework developed in this study provides a useful tool to investigate NOM–mineral interactions. Although our study employed goethite as a model mineral, the underlying principles, electrostatic interactions, ligand exchange, and heterogeneity, are fundamental to NOM–mineral interactions and are therefore expected to be broadly applicable to other metal (hydr)­oxides and mineral types. Nevertheless, the quantitative application of this framework requires mineral-specific characterization and parameterization. For instance, crystalline oxides such as hematite may follow a similar spatial limitation behavior as goethite, whereas poorly crystalline phases like ferrihydrite, with particle sizes comparable to NOM molecules and strong tendencies to aggregate or coprecipitate, may introduce additional complexities that warrant further investigation.^46,47^ The preferential adsorption and interfacial exchange of NOM studied in this paper can be further explored using ternary systems with oxyanions that can trace the interfacial ligand and charge distribution of NOM particles in more detail and, therefore, will be part of future work.

## Supplementary Material



## Notes

Details are physicochemical properties of HA and FA; size exclusion chromatography (SEC) measurements; pH–charge curves of HA and FA and fitting of the NICA–Donnan model; filtration recovery data for HA and FA; quantification of HA and FA using UV–vis spectroscopy and acid precipitation methods; particle-level heterogeneity probed by phosphate; adsorption energy change and adsorption affinity; spatial distribution functionals; model parameters for HA and FA adsorption to goethite; mean molar mass of total HA and FA; adsorption on molar mass; sensitivity analysis; electrostatic potential profile; and modeling NOM competition with phosphate (PDF)
