# Supplementary material for: Deciphering Competitive Interactions of Natural Organic Matter Components at Metal Oxides: Insights from Experiments and Modeling
Source: Environ Sci Technol. 2025 Oct 30;59(44):23849–58. doi: 10.1021/acs.est.5c00782 (PMC12613805; doi:10.1021/acs.est.5c00782)
Supplement: Supplementary file 1 [file es5c00782_si_001.pdf]

Supporting Information

Deciphering Competitive Interactions of Natural Organic Matter Components at Metal Oxides: Insights from Experiments and Modeling

Yun Xu[a,b], Tjisse Hiemstra[a], Yilina Bai [a,c], Wenfeng Tan[b], Liping Weng[a,d*]

[a] Soil Chemistry and Chemical Soil Quality Group, Wageningen University & Research, 6708 PB, Wageningen, the Netherlands

[b] State Environmental Protection Key Laboratory of Soil Health and Green Remediation, College of Resources and Environment, Huazhong Agricultural University, 430070, Wuhan, China

[c] Chongqing Academy of Agriculture Sciences, Chongqing 40000, China

[d] Agro-Environmental Protection Institute, Ministry of Agriculture, 300191, Tianjin, P. R. China

*Corresponding author: liping.weng@wur.nl

1. Physicochemical properties of HA and FA

The elemental composition of purified HA and FA was determined using freeze-dried subsamples on Organic Elemental Analyzer (Elementar Vario EL, Germany). UV light absorbance spectra were measured on UV-Visible Spectrophotometer (PN3212 UV Detector, Postnova Analytics, Germany), and the specific UV absorbance at 254 nm ($SUVA_{254}$) was calculated, which is related to the aromaticity of the samples. The weight-averaged molar mass (M_W) is measured with size-exclusion chromatography (SEC) using 0.1 M phosphate buffer (pH 6.9). Details for M_W determination can be found in the next section "size exclusion chromatography (SEC) measurements". The physicochemical properties of HA and FA can be found in Tables S1&S2 and Figure S2.

Table S1 Elemental compositions, $SUVA_{254}$ values and weight-averaged molar mass (M_W) of HA and FA

Samples	C %	H %	O %	N %	$SUVA_{254}$ (L mgC^{-1} m^{-1})	M_W kDa
HA	53.8	4.6	37.2	4.5	5.28	17
FA	49.6	4.6	38.1	7.7	6.90	1.8

2. Size exclusion chromatography (SEC) measurements

Size exclusion chromatograms of humic acid (HA), fulvic acid (FA), and their mixtures before and after adsorption were obtained with size exclusion chromatography and UV light absorption (SEC-UV). The mobile phase used was a 1:1 mixture of 0.1 M NaH_2PO_4 and 0.1 M Na_2HPO_4 (pH 6.9), flowing at a rate of 1.0 mL min^{-1}. A volume of 100 μL sample solution was injected. The UV absorption at 254 nm of the effluent was detected with a diode array detector (PN3241 UV Detector, Postnova Analytics, Germany) and was used for further data processing. The calibration curve for the molar mass calculation can be found in Figure S1. The mean molar mass (M_W) in the solution sample measured was determined with Eq S1.

$$M_w = \frac{\sum_{i=1}^{N} f_i M_i^2}{\sum_{i=1}^{N} f_i M_i} = \frac{\sum_{i=1}^{N} h_i M_i}{\sum_{i=1}^{N} h_i}$$

(S1)

where f_i is the frequency of the number of molecules, M_i is the mass of a characteristic molecule of fraction i, N is the number of molecular fractions according to the molar mass, and h_i is the absolute absorbance at 254 nm. The SEC chromatograms of HA and FA at the adsorbed phase were calculated from the difference in the chromatograms of solution samples before and after adsorption.

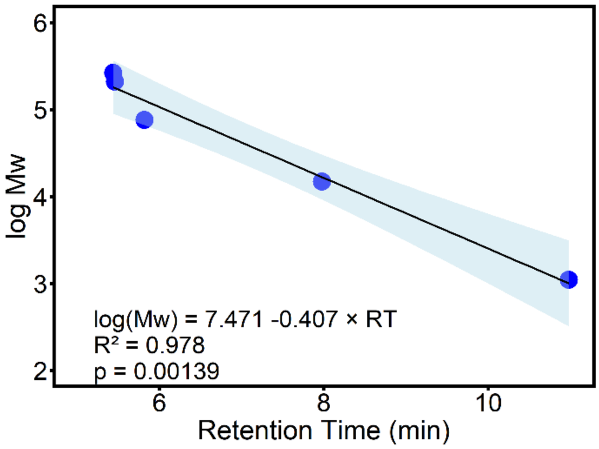

Figure S1. Semi-log calibration curve for the SEC measurements. Polystyrene sulfonates (PSS) particles with a molar mass of 1.3, 13, 80, 200, and 280 kDa (Sigma Aldrich, Buchs, Switzerland) were used to calibrate the molar mass over retention time. The light blue area represents the 95% confidence interval.

In principle, size exclusion chromatography discriminates based on particle size. We have used polystyrene sulfonates (PSS) to calibrate the relationship between the retention time and particle size. However, the manufacturer reported only a molar mass for PSS (800 kg m^{-3}). When using the calibration equation to calculate the molar mass of HA or FA, it implies that we assumed an equal mass density and hydrodynamic size for PSS and HA or FA. Using a density of 1250 kg m^{-3} for HA and FA, the calculated size is ~14 % smaller than using the density of 800 kg m^{-3} for PSS provided by the manufacturer. The calibration curve used for SEC measurements, along with its 95% confidence interval, is provided in Figure S1.

3. pH-charge curves of HA and FA and fitting of NICA-Donnan model

The pH-charge curve for humic acid (HA) used in current study was measured and reported by Deng et al.[1], whereas that of fulvic acid (FA) was determined in the current study, through acid-base titration following a method similar to that described by Tan

et al.[2]. Briefly, titrations were conducted at a concentration of 0.3 g L^{-1} FA in 0.01, 0.05, and 0.10 M NaCl under a N_2 atmosphere. For each ionic strength, a back-and-forth titration loop was performed between pH 3.2 and 11.0. The changes in charge were calculated from the titration data, where the Davies equation was used to determine the activity coefficient. The absolute charge was then derived by incorporating the initial charge of FA at the chosen reference point of pH 9.0 in 0.01 M NaCl, which was determined based on the pH and the amount of NaOH and HCl consumed during the preparation of the FA solution from the freeze-dried sample. Using the pH-charge data, the NICA-Donnan model parameters were optimized (Table S2). The pH-charge data and the NICA-Donnan model results are presented in Figure S2.

Table S2. NICA-Donnan model parameters for HA and FA

	Type of ligand	Ligand density Q_{max} (mol kg^{-1})	p	$\log\widetilde{K}_H$	n_H	$\log\widetilde{K}_{s,1}$	$n_{S,1}$	b
**HA**	Carboxylic	2.32	0.61	3.03	0.81	-1	0.81	0.49
	Phenolic	2.39	0.41	8.00	0.63			
**FA**	Carboxylic	5.91	0.64	2.40	0.66	-1	0.66	0.57
	Phenolic	1.86	0.67	8.60	0.76			

- The NICA-Donnan parameters ($Q_{max,1}$, $\log\widetilde{K}_1, p_1, p_2$) have been derived by fitting the model to the pH-charge curves of the HA and FA under study, while keeping the other model parameters as the generic HA or FA parameters[3,4]. The parameters for HA were fitted by Deng et al.[1], whereas the parameters for FA were fitted in the current study.
- Q_{max} and p are respectively the site density and heterogeneity parameters. $\log\widetilde{K}_H$ and n_H are respectively the median affinities and non-ideality parameters for the protonation reactions. $\log\widetilde{K}_{s,1}$ and n_S are respectively the median affinities and non-ideality parameters for the formation of innersphere complexes between the carboxylic groups in the first Stern layer with the singly coordinated sites on goethite. The values of $\log\widetilde{K}_{s,1}$ were adopted from Weng et al.[5], and the value of n_S is assumed equal to n_H[6]. b is an empirical parameter describing the dependence of Donnan volume on ionic strength, taken from the G-FA dataset of Milne et al.

(2001), although the implied volumes are not fully consistent with actual FA molecular densities.

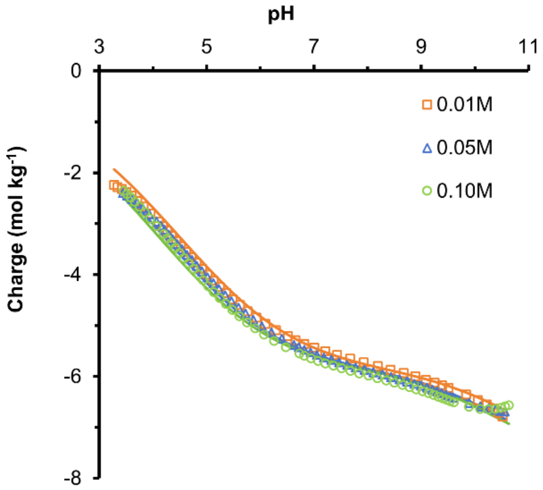

Figure S2. pH-charge curves of FA at three ionic strengths (0.01, 0.05 and 0.10 M NaCl). Symbols are experimental data, and lines are NICA-Donnan model calculations.

4. Filtration recovery data for HA and FA

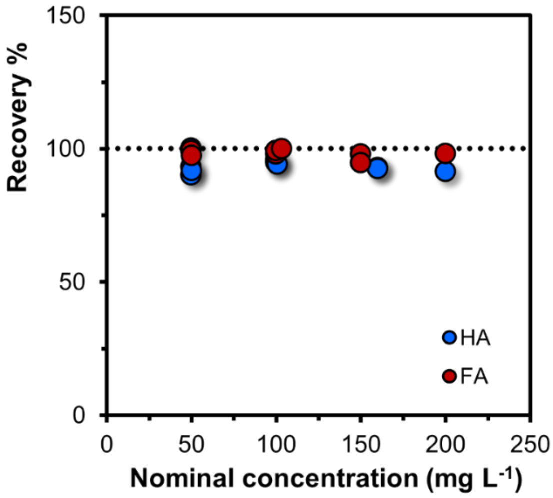

Figure S3. Filtration (0.22-μm filters) recovery data for HA and FA in the absence of goethite. Recovery of HA (blue circles) and FA (red circles) at different nominal concentrations ranging from 0 to 200 mg L^{-1}, at pH 5~6, 0.01 M NaCl.

5. Quantification of HA and FA using UV–Vis spectroscopy and acid precipitation methods

We applied the UV–Vis spectroscopy method developed by Xu et al.[7] to quantify humic acid (HA) and fulvic acid (FA) concentrations in their mixtures. Xu et al.[7] have

demonstrated that this approach remains effective even after adsorptive fractionation onto goethite, due to the characteristic differences in the spectral shapes of HA and FA.

UV-Vis light absorbance spectrum was collected on the test solution over the wavelength of 230-600 nm on a spectrophotometer (PN3212 UV Detector, Postnova Analytics, Germany). The total organic carbon content (TOC) of the sample was also measured with a TOC analyzer (Sievers 900, GE, USA) after adjusting the solution pH to approximately 6.0. For quantifying HA and FA concentrations in their mixtures after adsorptive fractionation, we used the following equation:

$$\mathrm{TS}_{\mathrm{Mix}\,(i)\,(\mathrm{model})} = A\ \mathrm{R}_{\mathrm{HA}(i)} + B\ \mathrm{R}_{\mathrm{FA}(i)} \qquad \text{(S2)}$$

in which $\mathrm{TS}_{\mathrm{Mix}(i)(\mathrm{model})}$ ($\mathrm{L \cdot mgC^{-1} \cdot cm^{-1}}$) is the modeled carbon-normalized absorbance of the HA–FA mixture at wavelength *i*; $\mathrm{R}_{\mathrm{HA}(i)}$ and $\mathrm{R}_{\mathrm{FA}(i)}$ represent the carbon-normalized absorbance of original HA and FA ($\mathrm{L \cdot mgC^{-1} \cdot cm^{-1}}$) at wavelength *i*; *A* and *B* are parameters related to the relative concentration of HA and FA. Values of *A* and *B* were obtained by fitting the equation to the measured UV-Vis spectrum through nonlinear regression, minimizing the sum of squared residuals between measured and modeled absorbance values over the whole wavelength range (230-600 nm). From the fitted parameters, the fraction of HA and FA in the mixture after fractionation is calculated as $f_{\mathrm{HA}} = A/(A+B)$, and $f_{\mathrm{FA}}=1-f_{\mathrm{HA}}$. These fractions can be combined with the TOC analysis of the mixture ($\mathrm{TOC_{total}}$, $\mathrm{mgC\ L^{-1}}$) to derive the concentrations of HA (C_{HA}) and FA (C_{FA}):

$$C_{\mathrm{HA}} = f_{HA} \times \mathrm{TOC_{total}} \qquad \text{(S3a)}$$

$$C_{\mathrm{FA}} = (1-f_{HA}) \times \mathrm{TOC_{total}} \qquad \text{(S3b)}$$

For comparison, the acid precipitation method was also employed. This classical approach separates HA and FA based on their solubility at low pH: HA precipitates at pH 1.0 while FA remains in solution. In this study, 10 mL of the test solution was acidified to pH 1.0 using 6 M HCl and allowed to stand for 20 hours to promote aggregation and settling. The sample was then centrifuged (20 min at 18,000 g), and the supernatant was filtered through a 0.45 μm membrane. The filtrate was neutralized to approximately pH 6.0 using NaOH before TOC analysis ($\mathrm{TOC_{filtrate}}$) with a TOC analyzer (Sievers 900, GE, USA). The difference between the original TOC content ($\mathrm{TOC_{total}}$) and $\mathrm{TOC_{filtrate}}$ will be equal to the carbon concentration of HA ($C_{\mathrm{HA}}=\mathrm{TOC_{total}}$

– $TOC_{filtrate}$), whereas C_{FA}=$TOC_{filtrate}$.

In this study, the final HA and FA concentrations were obtained by averaging the values measured with the UV–Vis and acid precipitation methods.

6. Particle-level heterogeneity probed by phosphate

In our previous work, the particle-level heterogeneity parameter (q in Eq 1) in the adsorption affinity of HA to goethite was probed with phosphate (PO_4)[8]. The particle-level heterogeneity of FA can be determined similarly by fitting the adsorption of FA and PO_4 to goethite. In the current study, a batch adsorption experiment was carried out, in which the phosphate addition (x = 0 – 0.45 mM phosphate as $NaH_2PO_4{\cdot}2H_2O$, expressed as $x$$PO_4$) was varied at a constant FA addition (y = 150, or 200 mg L^{-1}, noted as $x$$PO_4$ + [yFA]) at pH 4. The adsorption of FA and PO_4 are given in Figure S4.

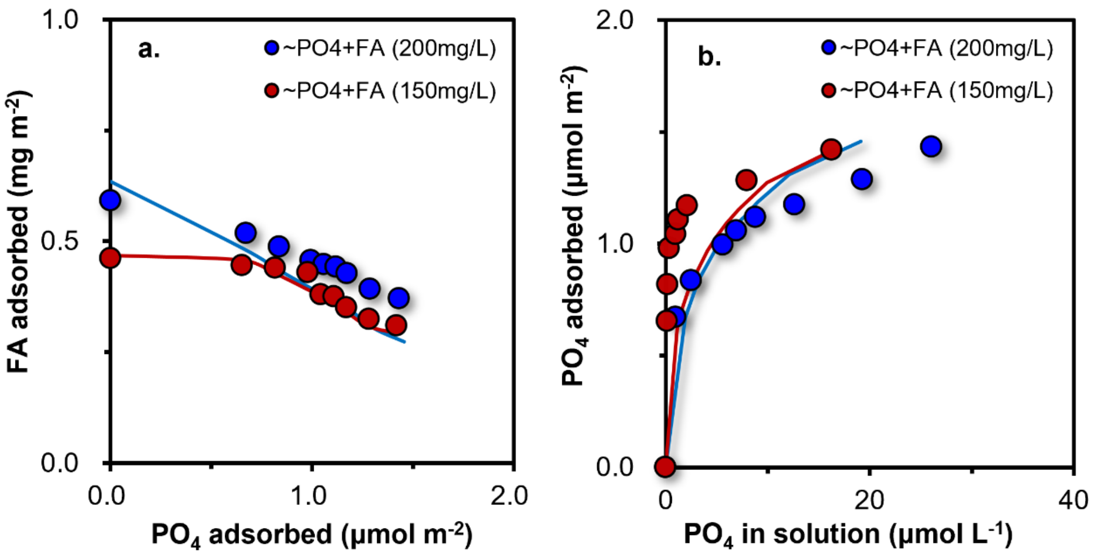

Figure S4. (a.) Adsorbed FA versus the phosphate surface loading on goethite at pH 4. (b.) Adsorption isotherms of PO_4 in the presence of two levels of FA. The adsorption system contains 3 g L^{-1} goethite and 0.01 M NaCl. The PO_4 addition varied from 0 to 0.45 mM at two fixed FA levels of 150 (red) and 200 (blue) mg L^{-1}. Symbols: experimental data; Solid lines: LCD_{cc} model prediction considering the particle heterogeneity (fitted q = 0.3).

The affinity of NOM adsorbing to metal (hydr)oxides is related to a range of factors. Primary factors are the molar mass and size as well as the density and acidity of the functional groups. To derive the particle-level heterogeneity parameter, using the

same approach applied to HA in our previous work[8], we found that our data for FA adsorption can be best described using the LF approach (Eq 1, main text) with $q = 0.3$[9].

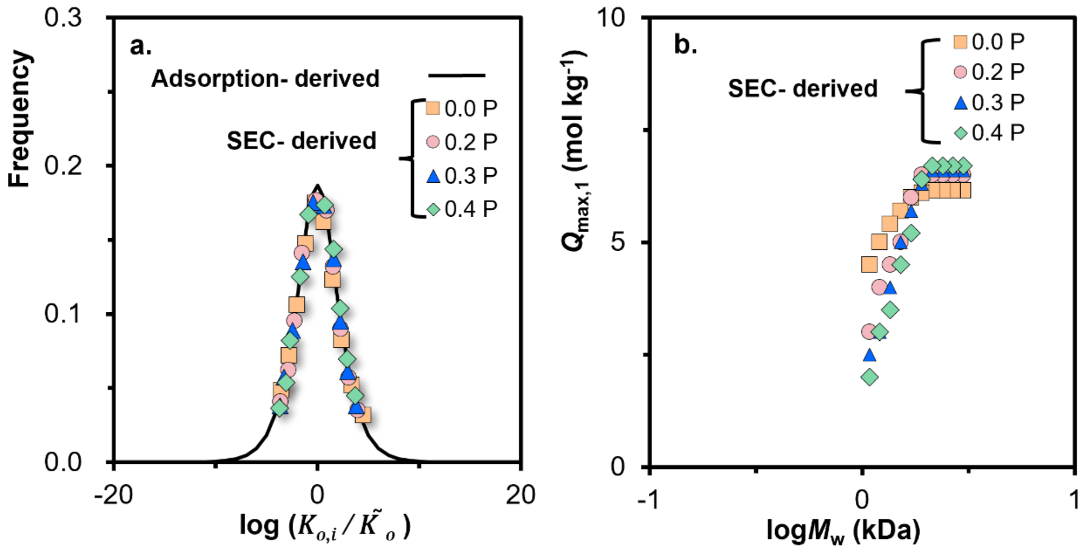

Figure S5. (a) Relative affinity distribution according to FA adsorption data in 0.01 M NaCl interpreted with LF model (Eq 1, main text) using $q = 0.3$ (full line) compared to the calculated affinity distribution using the molar mass distribution measured with SEC for adsorption series of xPO_4+[200FA] in combination with an adaptation for each mass fraction of the carboxylic group density $Q_{max,1}$ (symbols). (b) Relationship between the optimized carboxylic group density ($Q_{max,1}$) and the SEC-measured molar mass (logM_w) of the adsorbed FA. The notation 0, 0.2, 0.3, and 0.4 P refers to the total concentration of phosphate added in mM in the adsorption series of xPO_4+[200FA].

The heterogeneity in the affinity of FA can be well described (Figure S5a) using measured M_w distribution of adsorbed FA while fitting the variation in site density of the carboxylic groups ($Q_{max,1}$). For FA, a positive correlation between these fitted carboxylic group densities and molar mass is found (Figure S5b), which indicates that smaller-sized FA particles in the distribution have a lower carboxyl density than larger FA particles. The combination of M_w and $Q_{max,1}$ determines the number of carboxylic ligands per NOM particle (N_{max}), which is a key parameter for the specific adsorption affinity K_{sp} according to the ADAPT model presented in Section 7 of Supporting Information (Eq S4a).

7. Adsorption energy change and adsorption affinity

In the LCD modeling, the median adsorption affinity ($\widetilde{K}_{\mathrm{o}}$) calculated with the ADAPT module has a non-specific and specific energy contribution[6]. The non-specific energy contribution ($\Delta F_{\mathrm{p,nsp}}$) is related to removing the counter ions (e.g. Na^{+}) from NOM in the solution phase before placement of this hypothetical NOM* into the adsorbed phase. The specific energy contribution ($\Delta F_{\mathrm{p,sp}}$) originates from the free energy change of NOM when the ligand speciation of the NOM changes upon adsorption. Upon adsorption, the NOM* (without excess counter ions) enters the electrostatic field of the metal (hydr)oxide surface and may change in charge and corresponding chemical potential due to protonation or deprotonation reactions on the functional groups and formation of innersphere surface complexes. When translated to reaction constants, the specific ($\widetilde{K}_{\mathrm{p,sp}}$) and non-specific ($\widetilde{K}_{\mathrm{p,nsp}}$) part can be expressed as[6]:

$$\widetilde{K}_{\mathrm{p,sp}} = \prod_{l} \prod_{j=1}^{2} \left\{ \left(\frac{1-\sum \theta_{i,j,\mathrm{sol}}}{1-\sum \theta_{i,j,\mathrm{ads},l}} \right)^{\frac{N_{\mathrm{max},j,l}}{n_{\mathrm{H},j}\ p_j}} \right\} \left(\frac{B_{\mathrm{D}}}{B_l} \right)^{N_{\mathrm{max},j,l}} \quad \text{(S4a)}$$

$$K_{\mathrm{p,nsp}} = e^{-\frac{\Delta F_{\mathrm{p,nsp}}}{\mathrm{R}T}} = e^{-\sum c_{i,0}\left(e^{-\frac{z_i \Psi_{\mathrm{D}}}{\mathrm{R}T}}-1\right)\ V_{\mathrm{D}} M_{\mathrm{w}}} \quad \text{(S4b)}$$

in which $N_{\mathrm{max},j,l}$ (mol mol^{-1}) is the number of type j ligands (j=1, carboxylic; j=2, phenolic) per NOM particle, located at a specific electrostatic plane of the interface, i.e. the surface (l=0), the first (l=1) and second (l=2) Stern plane, or at an additional plane of adsorption at a distance d outside the surface (l= d). The number of functional groups (mol mol^{-1}) per location l can be given as:

$$N_{\mathrm{max},j,l} = f_l\ Q_{\mathrm{max},j} M_{\mathrm{w}} \quad \text{(S4c)}$$

in which f_l is the fraction of the NOM attributed to an electrostatic plane l (see also Eq 2 in the main text), M_{w} (g mol^{-1}) is the mean molar mass of adsorbed NOM, and $Q_{\mathrm{max},j}$ (mol g^{-1}) is the site density of carboxylic or phenolic groups per mass of NOM. In Eq S4a, $n_{\mathrm{H},j}$, and p_j are the NICA parameters that can be found in Table S2; $\theta_{i,j,\mathrm{sol}}$ is the fraction of type j ligands of NOM that are complexed with component i (protons or other ions) in the solution phase; $\theta_{i,j,\mathrm{ads},l}$ is the fraction of type j ligands of adsorbed NOM that are complexed with component i (protons or surface sites) in layer (l); B_l is

the Boltzmann factor at each electrostatic plane where the charge of NOM is located; B_D is the Boltzmann factor for the Donnan phase of NOM in solution. In Eq S4b, $C_{i,0}$ (mol L^{-1}) is the concentration of the non-specifically bound counter ions with a charge (z_i) present in the Donnan phase with a volume V_D (L g^{-1}) and potential ψ_D (V).

8. Spatial distribution functionals

In the LCD_{cc} model, the spatial distribution of adsorbed NOM particles (f_{0+1}, f_{1+2}, f_d) is indispensable in calculating the adsorption affinity (Eq S5a-b). The spatial distribution of NOM can be effectively described with the maximum occupation of the compact part of the EDL (θ_s) (Eq 2c, main text) and the relative ligand distribution (R) (Eq 2d, main text)[8]. Both conformational parameters can be linked to potential gradients, defined as:

$$\theta_S = \theta_{min} + \Delta\theta_{max} \frac{1}{1 + a\ e^{-b\ \Delta\psi_{02}}}$$

(S5a)

$$R = R_{min} + \Delta R_{max} \frac{1}{1 + c\ \ e^{-d\ \Delta\psi_{01}}}$$

(S5b)

where θ_{min} and R_{min} are respectively the minimum values of θ_s and R when the electrostatic potential gradients are very small; $\Delta\theta_{max}$ and ΔR_{max} are the maximum values of θ_s and R that are reached when the electrostatic field is highly attractive; $\Delta\psi_{02}$ and $\Delta\psi_{01}$ are gradients of electrostatic potential over 0- and 2-plane as well as 0- and 1-plane, respectively; a, b, c, and d are constants, determining the shape of the sigmoidal curves of θ_s and R.

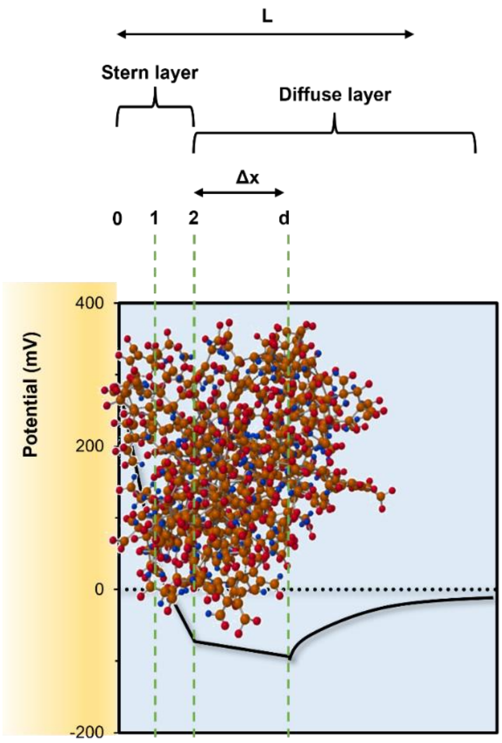

Figure S6. Schematic representation of the interfacial adsorption of a humic particle onto a metal (hydr)oxide surface in the electrical double layer (EDL). (figure adapted from Xu et al.[8])

9. Model parameters for HA and FA adsorption to goethite

Table S3. CD-MUSIC model parameters

**Specific surface area (BET-N_2) (m^2 g^{-1})**	**Capacitances of Stern layers (F m^{-2})**		**Site density (nm^{-2})**		**Protonation $\log K_{H1}=\log K_{H2}$**	**Ion pair formation**		**Charge distribution of $RCOO^-$ in the innersphere complex**	
	C_1	C_2	$\equiv FeOH^{0.5-}$	$\equiv Fe_3O^{0.5-}$		$\log K_{Na}$	$\log K_{Cl}$	Δz_0	Δz_1
**96**	0.83	0.74	3.45	2.7	9.3	-0.60	-0.45	-0.5	-0.5

- The parameters were adopted from Weng et al.[10] and Hiemstra and van Riemsdijk[11]

Table S4. Summary of parameters used in the modeling

Modules	Types of parameters		
	**Kept fixed as model input in the current study**		**Adjustable or iterated**
	From literature	Calculated	
**NICA-Donnan**	Q_{max}, p, $\log\widetilde{K}_{H}$, n_{H}, b (see Table S2)		
**NICA-LD**	Q_{max}, p, $\log\widetilde{K}_{H}$, n_{H}, $\log\widetilde{K}_{s,1}$, $n_{S,1}$ (see Table S2) charge distribution (Δz_0 and Δz_1) for innersphere complex (see Table S3)		
**CD-MUSIC**	Specific surface area of goethite, C_1, C_2, site densities of $\equiv FeOH^{0.5-}$ and $\equiv Fe_3O^{0.5-}$, $\log K_{H}$, $\log K_{Na}$, $\log K_{Cl}$, charge distribution (Δz_0 and Δz_1) for protonated sites and ions pairs (see Table S3)		
**ADAPT**	ρ_p (set to ρ_p = 1250 kg m^{-3})	M_w (derived from Eq 3, main text); q (probed by PO_4 in the single HA or FA adsorption systems)	spatial distribution (f_1, f_2, f_d) regulated by potential-gradient (Eq 2a-d, Eq S5a-b); volume of adsorbed HA or FA (Eqs 5-7, main text)

10. Mean molar mass of total HA and FA

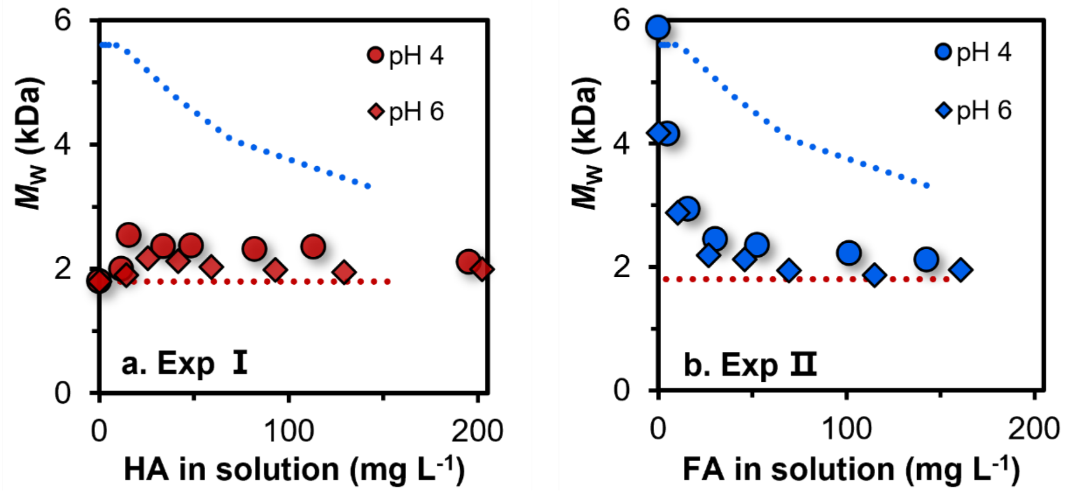

Figure S7. Mean molar mass (M_W) of total HA and FA in the adsorbed phase in Exp I (a.) and Exp II (b.). Exp I: FA addition was kept constant at 100 mg L^{-1} and HA addition varied from 0 to 240 mg L^{-1}. Exp II: HA addition was kept constant at 160 mg L^{-1} and FA addition varied from 0 to 200 mg L^{-1}. Both Exp I and II were conducted in 1 g L^{-1} goethite and 0.01 M NaCl at pH 4 (circles) and 6 (diamonds). The blue dotted line represents the high boundary of mean molar mass (M_w) derived from the single HA adsorption experiments that have the same level of adsorbed HA as in the binary FA/HA systems[12], whereas the red dotted line is the lower boundary derived from the single FA adsorption experiment, showing negligible changes in the mean molar mass of adsorbed FA.

11. Adsorption on molar mass

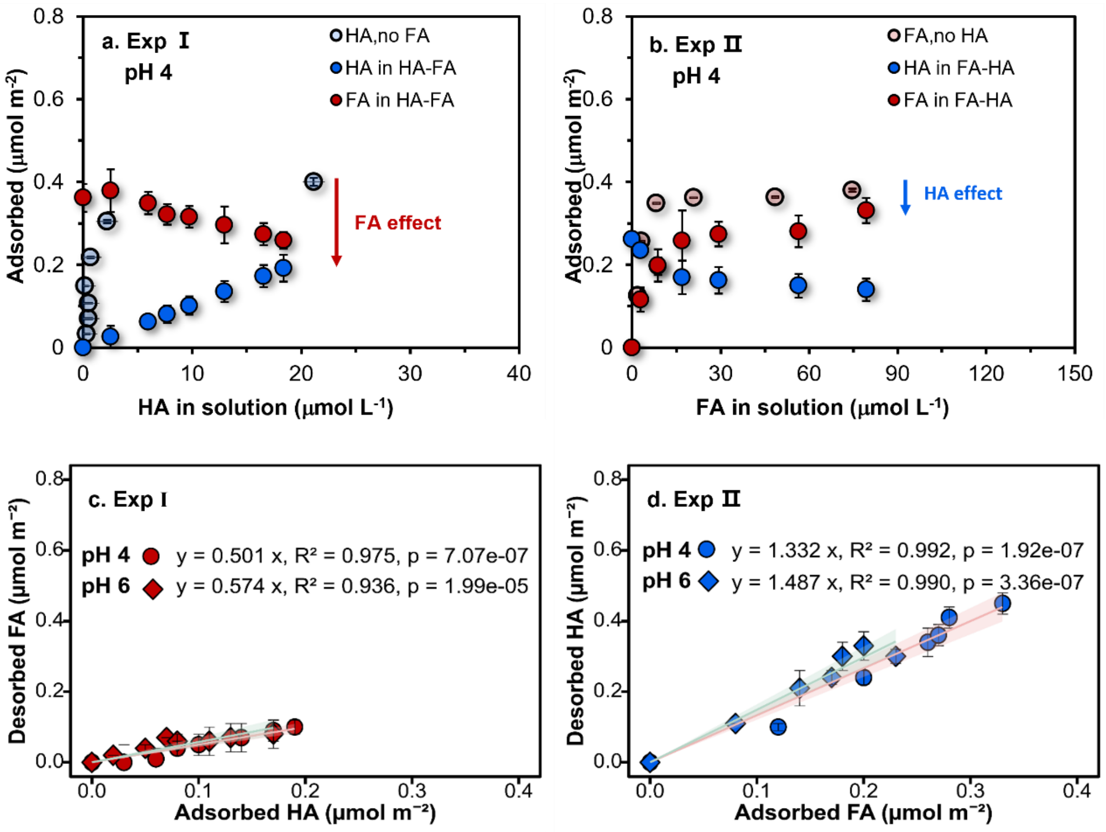

Figure S8. Competitive adsorption of HA and FA on goethite surface expressed on a molar basis at pH 4 with a background electrolyte of 0.01 M NaCl in Exp I (a.) and Exp II (b.). Desorbed FA related to adsorbed HA in Exp I (c.), and desorbed HA related to adsorbed FA in Exp II (d.) at pH 4 (circles) and 6 (diamonds). Error bars represent the standard deviation of duplicate measurements. Confidence intervals of the fitted regressions are shaded.

12. Sensitivity analysis

1) mass density (ρ_p)

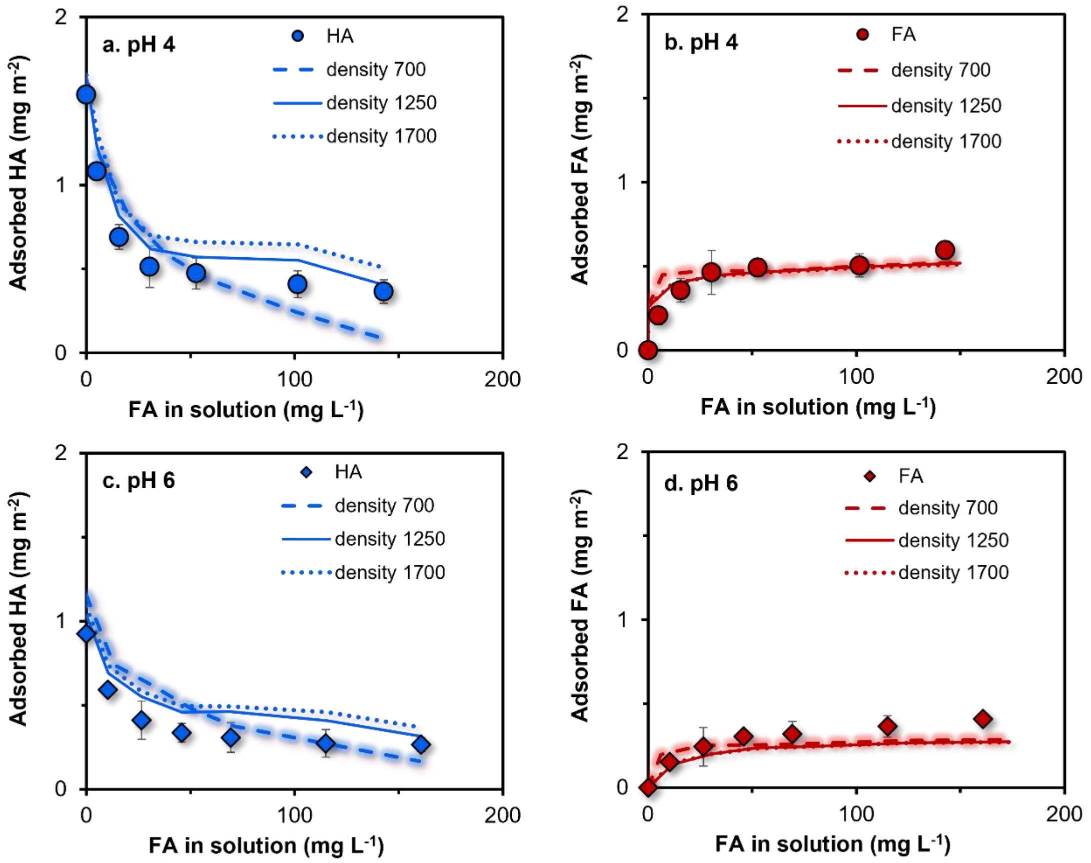

Figure S9. Sensitivity analysis of the modeled adsorption isotherms in Exp II to the assumed particle mass density (ρ_p) of HA and FA. In Exp II, HA concentration was fixed at 160 mg L^{-1}, while FA varied from 0 to 200 mg L^{-1} in 1 g L^{-1} goethite suspension with 0.01 M NaCl at pH 4 and 6. Dashed, solid, and dotted lines correspond to ρ_p value of 700, 1250, and 1700 kg m^{-3}, respectively, representing well-hydrated, intermediate, and dry particle state. FA adsorption is insensitive to ρ_p, whereas HA adsorption shows a moderate dependence.

2) Particle-level heterogeneity (q)

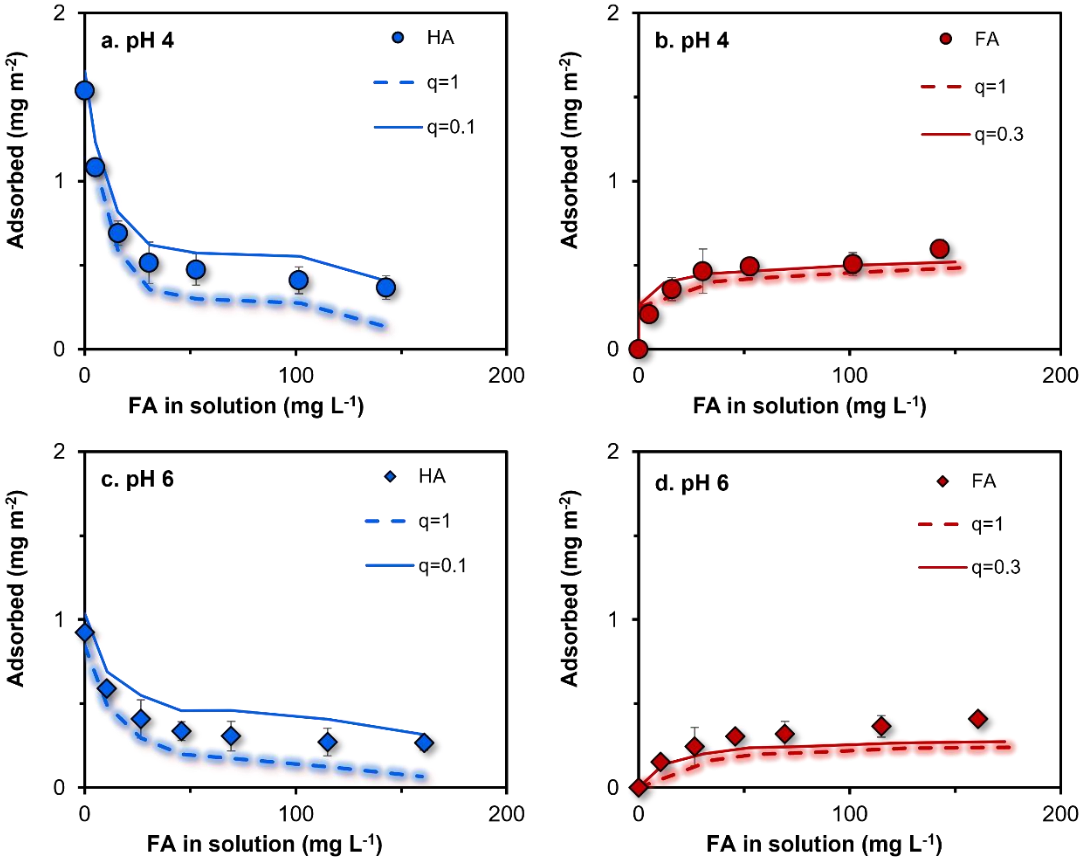

Figure S10. Sensitivity analysis of modeled HA and FA adsorption in Experiment II with respect to the assumed particle-level heterogeneity parameter (q). Solid lines: model simulations using previously constrained values probed by PO_4 adsorption, with q_{HA} = 0.1, q_{FA} = 0.3. Dashed lines: modeling without considering particle level heterogeneity (q= 1).

13. Electrostatic potential profile

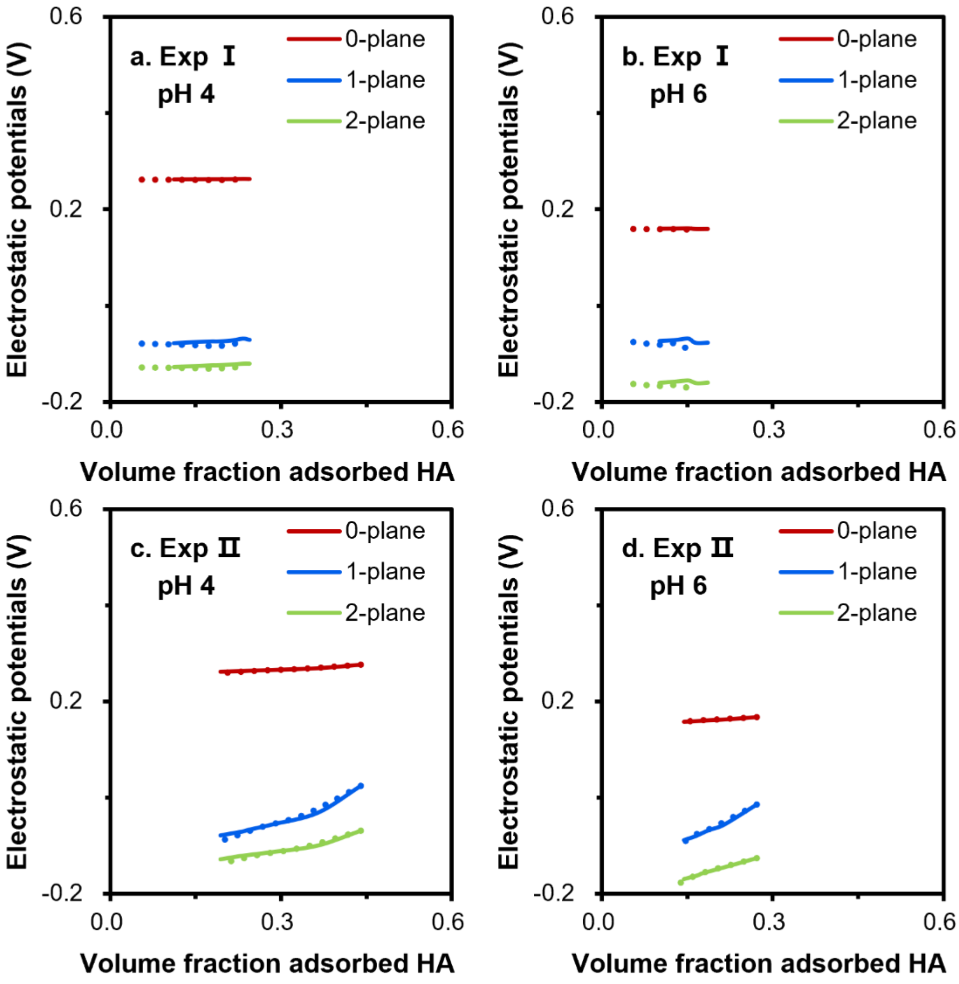

Figure S11. Calculated electrostatic potential at 0-, 1-, and 2-plane in the interface of goethite at pH 4 (a,c) and 6 (b,d) in 0.01 M NaCl as a function of the volume fraction occupied by adsorbed HA in Exp I and II, with constant or variable adsorption volume in the model. Exp I: FA addition was kept constant at 100 mg L^{-1} and HA addition varied from 0 to 240 mg L^{-1}. Exp II: HA addition was kept constant at 160 mg L^{-1} and FA addition varied from 0 to 200 mg L^{-1}. Dotted line: assuming relatively small space constraints (constant layer thickness (3 nm) and adsorption volume; solid lines: including stronger physical space limitation (variable layer thickness (0.8-3 nm) and adsorption volume).

14. Modeling NOM competition with phosphate

As Figure S12 illustrates, HA and FA compete differently with phosphate. At the same C and phosphate loadings, FA releases more phosphate than HA, particularly at low loadings. The stronger competition of FA, releasing more adsorbed phosphate, can be explained by the stronger electrostatic interactions between adsorbed FA particles and phosphate as a result of the higher functional group density of FA[4]. Additionally, the larger HA particles will change their conformation, particularly at a high surface loading, substantially increasing the proportion of functional groups that reside outside the compact region of the electrical double layer (EDL), thereby rendering them less effective in competing with phosphate.

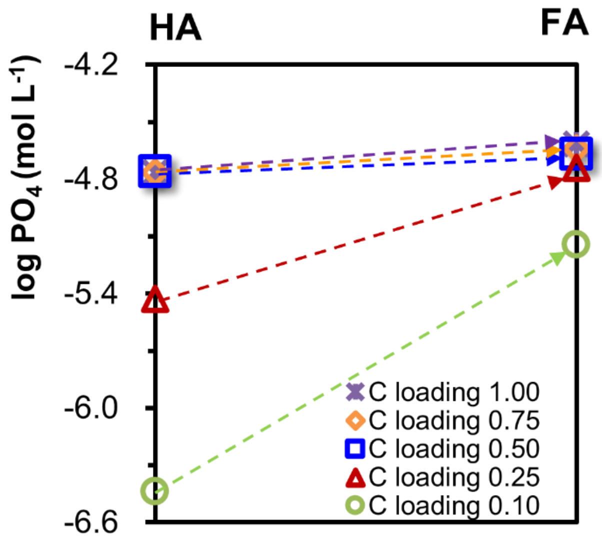

Figure S12. Modeled PO_4 equilibrium concentration illustrating the difference in the PO_4 competition of HA and FA at pH 4 in 0.01 M NaCl. Goethite 1 g L^{-1}. PO_4 surface loading was maintained at 1.52 μmol m^{-2}. The C loading of HA or FA added varied from 0.1 to 1.0 mg C m^{-2}. The carbon content of HA and FA used in the calculation is respectively 58% and 48%.

References

(1) Deng, Y.; Weng, L.; Li, Y.; Ma, J.; Chen, Y. Understanding Major NOM Properties Controlling Its Interactions with Phosphorus and Arsenic at Goethite-Water Interface. *Water Res.* **2019**, *157*, 372–380. https://doi.org/10.1016/j.watres.2019.03.077.

(2) Tan, W.; Xiong, J.; Li, Y.; Wang, M.; Weng, L.; Koopal, L. K. Proton Binding to Soil Humic and Fulvic Acids: Experiments and NICA-Donnan Modeling. *Colloid Surface A* **2013**, *436*, 1152–1158. https://doi.org/10.1016/j.colsurfa.2013.08.010.

(3) Milne, C. J.; Kinniburgh, D. G.; Tipping, E. Generic NICA-Donnan Model Parameters for Proton Binding by Humic Substances. *Environ. Sci. Technol.* **2001**, *35* (10), 2049–2059.

(4) Milne, C. J.; Kinniburgh, D. G.; van Riemsdijk, W. H.; Tipping, E. Generic NICA−Donnan Model Parameters for Metal-Ion Binding by Humic Substances. *Environ. Sci. Technol.* **2003**, *37* (5), 958–971. https://doi.org/10.1021/es0258879.

(5) Weng, L.; Van Riemsdijk, W. H.; Hiemstra, T. Humic Nanoparticles at the Oxide−water Interface: Interactions with Phosphate Ion Adsorption. *Environ. Sci. Technol.* **2008**, *42* (23), 8747–8752. https://doi.org/10.1021/es801631d.

(6) Weng, L.; Van Riemsdijk, W. H.; Koopal, L. K.; Hiemstra, T. Ligand and Charge Distribution (LCD) Model for the Description of Fulvic Acid Adsorption to Goethite. *J. Colloid Interface Sci.* **2006**, *302* (2), 442–457. https://doi.org/10.1016/j.jcis.2006.07.005.

(7) Xu, Y.; Bai, Y.; Hiemstra, T.; Tan, W.; Weng, L. Resolving Humic and Fulvic Acids in Binary Systems Influenced by Adsorptive Fractionation to Fe-(Hydr)Oxide with Focus on UV−Vis Analysis. *Chem. Eng. J.* **2020**, *389*, 124380. https://doi.org/10.1016/j.cej.2020.124380.

(8) Xu, Y.; Bai, Y.; Hiemstra, T.; Weng, L. A New Consistent Modeling Framework for the Competitive Adsorption of Humic Nanoparticles and Oxyanions to Metal (Hydr)Oxides: Multiple Modes of Heterogeneity, Fractionation, and Conformational Change. *J. Colloid Interface Sci.* **2024**, *660*, 522–533. https://doi.org/10.1016/j.jcis.2024.01.078.

(9) Xu, Y.; Bai, Y.; Hiemstra, T.; Weng, L. Ligand and Charge Distribution Modeling of Natural Organic Matter Adsorption on Metal (Hydr)Oxides: State-of-the-Art. *Rev Mineral Geochem* **2025**, *91A* (1), 229–250. https://doi.org/10.2138/rmg.2025.91A.07.

(10) Weng, L.; Van Riemsdijk, W. H.; Hiemstra, T. Adsorption of Humic Acids onto Goethite: Effects of Molar Mass, pH and Ionic Strength. *J. Colloid Interface Sci.* **2007**, *314* (1), 107–118. https://doi.org/10.1016/j.jcis.2007.05.039.

(11) Hiemstra, T.; Van Riemsdijk, W. H. On the Relationship between Charge Distribution, Surface Hydration, and the Structure of the Interface of Metal Hydroxides. *J. Colloid Interface Sci.* **2006**, *301* (1), 1–18. https://doi.org/10.1016/j.jcis.2006.05.008.

(12) Xu, Y.; Hiemstra, T.; Tan, W.; Bai, Y.; Weng, L. Key Factors in the Adsorption of Natural Organic Matter to Metal (Hydr)Oxides: Fractionation and Conformational Change. *Chemosphere* **2022**, *308*, 136129. https://doi.org/10.1016/j.chemosphere.2022.136129.
